# “Here’s Some Money, Your Work’s So Worthy?” A Brief Report on the Validation of the Functional Meaning of Cash Rewards Scale

**DOI:** 10.3389/fpsyg.2022.821501

**Published:** 2022-04-04

**Authors:** Anaïs Thibault Landry, Konstantinos Papachristopoulos, Marc-Antoine Gradito Dubord, Jacques Forest

**Affiliations:** ^1^John Molson School of Business, Concordia University, Montreal, QC, Canada; ^2^Athens School of Fine Arts, Athens, Greece; ^3^Département de Psychologie, Université du Québec à Montréal, Montreal, QC, Canada; ^4^École des Sciences de la Gestion, Université du Québec à Montréal, Montreal, QC, Canada

**Keywords:** self-determination, motivation, rewards, functional meaning, scale validation

## Abstract

In the present research, we validated a new scale developed from self-determination theory (SDT) to assess the functional meaning of cash rewards offered in the workplace. According to SDT, rewards can take on different meanings based on the way they are perceived by individuals. In a series of three studies in different socioeconomic contexts, we replicated the two-factorial structure of the scale measuring respectively workplace cash rewards’ informative and controlling meanings. In Study 1, we validated the English version of the scale by exploring and then confirming its two-factor structure with two English-speaking employee samples. We further replicated its two-factor structure in a French-speaking employee sample of employees in Study 2 and in a Greek-speaking employee sample in Study 3, allowing us to validate its French and Greek version. Results from our three studies show how distinct meanings attributed to cash rewards, i.e., informative or controlling, relate differently to autonomous and controlled forms of motivation based on SDT. These findings suggest that workplace cash rewards differently influence employees’ motivation depending on whether they are perceived as informative or controlling, thus providing empirical evidence for the theoretical and practical implications of SDT’s concept of functional meaning of cash rewards. Our research contributes to the assessment and understanding of employees’ experience of workplace cash rewards and provides empirical evidence that the concept of the functional meaning of cash rewards is a distinct concept from other money-related concepts such as subjective pay satisfaction, performance-contingent rewards, and financially contingent self-worth.

## Introduction

The relationship between rewards and work outcomes (e.g., motivation and performance) has long been a matter of debate and research in organizational settings and academia. This discussion has lately been further reinforced since employment relationships are undergoing significant changes and challenges (i.e., new types of contracts, volatile and uncertain social environment, and gig workers) and employees, apart from been solely paid, seem to care more systematically about meaningful aspects at work (e.g., relationships and social impact) while the new trends on the job market have gradually turned to an intrinsic focus on incentives and motivation (Chen and Hsieh, 2006), making the use of motivational theories to understand the meaning employees give to their monetary rewards and its impact on their behavior even more relevant (Zhang et al., 2018).

The companies’ decision to spend a major portion of their operating budget on compensation (salary, benefits, bonuses, etc.) as they seek to attract, motivate, and retain top performing employees (Delery and Roumpi, 2017; Patnaik and Suar, 2019), is partially supported by decades of research showing that financial incentives enhance performance (Bonner et al., 2000; Garbers and Konradt, 2014). Nevertheless, while earning a salary is fundamentally the basic premise for employment and monetary rewards are undeniably an important aspect of any employment relationship, such extrinsic monetary rewards have been critiqued for their “hidden costs” such as decline in the quality of the services provided (Qian and He, 2018), reduced volitional behavior of intrinsically motivated workers (Frey and Jegen, 2001; Georgellis et al., 2011; Gerhart and Fang, 2015), and lower satisfaction with the work itself – especially when such rewards are provided “ex-ante” for attaining specific standards of performance (Balkin et al., 2015). This is also mirrored on field research since human resource managers and reward professionals look quite often puzzled by the failure of reward strategies to increase productivity, boost job satisfaction, and enhance company performance (Kornelakis, 2018).

The current state of the empirical literature, therefore, does not allow us to draw strong generalizable conclusions. How can we actually reconcile these conflicting views? Why do we find these discrepancies in previous research? In answering these questions, we believe the missing piece is a discussion on how cash rewards can be perceived differently and thus take on different meanings for employees in the workplace, resulting in the seemingly disparate findings among previous literature on motivation. This might be one overriding weakness of extant research and their subsequent impact on motivation since this will likely depend on the interplay between all of these dynamics. In order to provide a more nuanced explanation of these different reward effects, this paper draws on the logic of a more comprehensive theory of motivation, known as self-determination theory (SDT; Deci, 1975; Ryan and Deci, 2000, 2017).

### Functional Meaning of Cash Rewards

Self-determination theory is an empirically based theory that focuses on different types, rather than amount, of human motivation (Deci and Ryan, 2008) and in recent years, has been increasingly applied to the work setting and to the topic of compensation (Gagné and Forest, 2008; Moller and Deci, 2014; Olafsen et al., 2015; Deci et al., 1999; Thibault-Landry et al., 2017, 2019a). According to SDT’s proposition, there are main two types of motivation: autonomous and controlled motivation. When people are autonomously motivated, they experience volition in their actions and integrate the activities into their sense of self. In contrast, when people experience controlled motivation, they feel pressured to act in certain ways as a function of external contingencies, such as rewards and punishment (Deci and Ryan, 2000, 2008). This concept is useful for understanding how different contextual factors can influence motivation in different ways. An important contribution of this theory is the proposition that extrinsic motivation need not be an invariably controlled form of motivation and that individuals can, under certain optimal conditions, fully internalize the value of their external experiences and behaviors (Deci and Ryan, 1985; Ryan and Connell, 1989). In line with SDT, external factors like cash rewards can take on different meanings depending on how they are perceived by individuals (Deci et al., 1989, 1994), which can lead individuals to adopt either autonomous or controlled motivation. Specifically, rewards take on an *informative* meaning when they are perceived as supportive and encouraging of individuals’ participation in their work, leading to autonomous motivation (Deci et al., 1989, 1994; Moller and Deci, 2014), whereas rewards take on a *controlling* meaning when they are presented as oppressive and constraining as to control individuals’ behavior and lead to controlled motivation (Deci et al., 1989, 1994). These two meanings are coined as the “functional meaning of rewards” in the SDT literature. Therefore, the underlying meaning of cash rewards, *per se*, may be what matters most when investigating the positive or negative influence of workplace cash rewards on employees’ motivation.

Supporting evidence for this claim has been found in other domains like education, sports, and health. For example, in the educational setting, students experience intrinsic enjoyment and enhanced academic performance when rewards and feedback are presented in supportive and encouraging ways (Koestner et al., 1984; Grolnick and Ryan, 1989; Williams and Deci, 1996; Black and Deci, 2000; Joussemet et al., 2004; Soenens et al., 2006). Informative feedback and rewards are also positively correlated with patients’ treatment adherence in health sectors (Williams et al., 1998) and with athlete training in sports settings (Bartholomew et al., 2010, 2011).

Research has shown that monetary rewards could have different functional meaning. Monetary rewards can be perceived as informative when presented in a supportive way as to encourage individuals’ efforts and participation in the activity, and convey appreciation and acknowledgment of their contribution. Thus, when monetary rewards are perceived as informative, they may positively contribute to employees’ psychological needs (Gagné and Deci, 2005; Tremblay et al., 2009; Moller and Deci, 2014; Thibault-Landry et al., 2017, 2019a). Conversely, monetary rewards can be perceived as controlling when presented in an oppressive, constraining way that pressures employees and emphasizes the performance goals to reach (Deci et al., 1989; De Cooman et al., 2013; Kuvaas et al., 2018). Hence, when monetary rewards are perceived as controlling, they may hamper psychological needs.

Intrigued by the distinct downstream implications of monetary rewards, Thibault-Landry et al. (2017, 2019a, 2019b) empirically tested these SDT-postulates, exploring the functional meaning of monetary rewards, and investigating how and why monetary rewards perceived as having an informative meaning and those perceived as having a controlling meaning could lead to different employee psychological experiences and functioning at work. They found empirical evidence that when it comes to informative monetary rewards, perceiving monetary rewards as informative appeared to be associated not only with feeling meaningfully connected, competent, and (even more so) autonomous at work, but could potentially buffer against feelings of incompetence, oppression, and rejection in the workplace, thus leading to optimal functioning at work (Thibault-Landry et al., 2019a). When it comes to controlling rewards, their results suggest that perceiving monetary rewards as controlling is associated with more negative psychological experience in the workplace, beyond feeling restricted, to feeling actively pressured and coerced into behaving in certain ways at work, thus leading to suboptimal functioning at work (Thibault-Landry et al., 2019a). As explained by Ryan and Deci (2008), “performance-contingent rewards have a strong risk of having controlling functional significance insofar as one feels pressured to meet an externally specified standard to get the reward” (p. 133). This type of controlling significance implies that employees no longer feel in control of the tasks that they consider important, but rather begin to shift their attention to those tasks that would dictate their reward allocation.

It is also interesting to note that Thibault-Landry et al. (2017, 2019a) found evidence of a significant association between the functional meaning of monetary rewards and employees’ psychological needs, including specifically the need for relatedness. This suggests that monetary rewards can have an effect on one’s sense of belonging and connection to others at work. This is consistent with past research findings linking monetary rewards with collaboration and teamwork, helping explain why under some circumstances compensation can hinders cooperation. That is, when perceived as controlling, monetary rewards can be associated with less cooperation amongst peers and colleagues, as well as a lower likelihood of engaging in prosocial behaviors like helping others. Further corroborating this, Papachristopoulos and Xanthopoulou (2019) found that relatedness need satisfaction moderated the relation between informative and controlling meaning of rewards and deviant behavior in a way that both meanings of monetary rewards related positively to deviant behavior under conditions of low relatedness need satisfaction, while they were unrelated to deviant behavior under conditions of high relatedness need satisfaction.

This emphasizes the point that offering a monetary reward, as well as any other type of rewards, occurs within the context of a social exchange between a giver and a recipient, and that as a result, this reward can take on different meanings for the recipients based on their perceptions of the giver’s intention. More specifically, together, these findings suggest that it is the functional meaning (informative or controlling) and the recipient’s interpretation of the reward, not the reward itself, which can have motivating or de-motivating effect (Thibault-Landry et al., 2017, 2019b). Hence, for monetary rewards to be efficient tools to motivate employees in healthy and optimal ways, there must be a genuine intent on behalf of the giver (i.e., the employer or the manager), and such intent must be perceived by the recipient (i.e., the employees).

### The Present Research

Although much research suggests that the functional meaning of cash rewards could lead to different types of motivation and experiences in the workplace, no scale to date has been developed to measure the two meanings of cash rewards proposed by SDT in the workplace. Therefore, our goal was to elaborate and validate a scale measuring the two meanings of cash rewards, namely, the informative and controlling meaning, according to SDT. Through three independent studies, we validate the underlying construct of the scale in three languages (English, French, and Greek). Then, we demonstrate the convergent and divergent validity as well as the concurrent validity of the scale.

#### Convergent/Divergent Validity

Recent reviews considering the application of SDT in the context of work organizations (e.g., Deci et al., 1999; Gagné and Deci, 2005; Gerhart and Fang, 2015) acknowledge that specific aspects of the social environment are central to facilitating basic need satisfaction and intrinsic motivation in the workplace. We thus provide evidence of the distinctiveness of the two functional meanings, i.e., the informative and the controlling meanings, of cash rewards by showing how they related to different types of motivation. We hypothesized that given that cash rewards are in and of themselves considered external factors, both informative and controlling meaning of cash rewards would be positively associated with controlled types of motivation—specifically—extrinsic and introjected motivation.

**Hypothesis 1:** Both informative and controlling meanings will be positively associated with extrinsic and introjected motivation.

However, the unique and original contribution of the functional meaning of rewards should lie in the perceived intent of using such reward. Hence, the underlying meaning of the cash rewards is hypothesized to be more specifically relevant with regards to autonomous types of motivations, such that the informative meaning of cash rewards will be positively associated with identified and intrinsic motivations, whereas the controlling meaning of cash rewards will be negatively associated with such types of motivation.

**Hypothesis 2:** Informative meaning of cash rewards will be positively associated with autonomous motivation, whereas controlling meaning of cash rewards will be negatively associated with autonomous motivation.

#### Concurrent Validity

Finally, to test the concurrent validity of the scale, we measure three different money and reward-related constructs. We show that the informative and controlling meaning of cash rewards are related, though not completely overlapping, with these constructs. In doing so, we illustrate how the functional meaning of cash rewards taps into a different construct than employees’ subjective pay satisfaction, financial contingent self-worth, and perceptions of the performance contingencies of the cash rewards used in their workplace.

With regards to subjective pay satisfaction, we include all four dimensions, namely satisfaction with pay level, benefits, pay raise, and pay structure (Judge, 1993), as it is reasonable to expect employees’ satisfaction with their work compensation to be related to some degree to their perception of the financial incentives and cash rewards used in their workplace.

In addition, we also examine financial self-worth, which is defined as the extent to which employees’ self-worth is contingent upon their ability to achieve financial success (Park et al., 2017). We chose to include financial self-worth since we hypothesized that employees’ assessment of their financial worth was likely to be related to some degree to the way they perceive financial incentives and cash rewards offered in their workplace.

Lastly but importantly, we measure and investigate the extent to which employees’ perceive the cash rewards offered in their workplace to be contingent on performance. We hypothesized this construct to be strongly associated, yet still conceptually distinct than their perception of their employer’s intention when using such rewards. As such, we did expect some degree of overlap since perceiving some degree of contingency is intricate to the notion of using cash to reward performance. In this sense, for cash rewards to hold different underlying meanings (informative vs. controlling), we assume that employees must perceive to a certain degree that cash rewards are tied to their work performance and used with this intent by employers.

**Hypothesis 3a:** The informative and controlling meanings of cash rewards will be moderately positively associated with dimensions of pay satisfaction, financially contingent self-worth and perception of the performance contingencies of the cash rewards.

**Hypothesis 3b:** The informative and controlling meanings of cash rewards will be more strongly associated with perception of the performance contingencies of the cash rewards than with dimensions of pay satisfaction and financially contingent self-worth.

### Development of the Functional Meaning of Cash Rewards Scale

Through an extensive literature review, we found two existing scales that could be used to measure the functional meaning of cash rewards as described in SDT. The two scales respectively closely covered the two functional meanings—controlling and informative—of rewards, as laid out by SDT. First, we look at the Controlling Use of Rewards subscale from the Controlling Coach Behavior Scale (Bartholomew et al., 2011). In the sports setting, this 3-item subscale is used to measure the extent to which coaches use external rewards to motivate their athletes (e.g., “My coach tries to motivate me by promising to reward me if I do well”). Second, we found the Perceived Autonomy Support Scale for Exercise Settings (PASSES; Hagger and Chatzisarantis, 2007). PASSES was used to measure the perception of autonomy support in exercise settings in terms of the instructors and was developed by Hagger et al. (2007). The items are scored on a seven-point Likert scale ranging from 1 (strongly disagree) to 7 (strongly agree). It is unidimensional and consists of 12 items (e.g., my health-related exercise instructor encourages me to engage in active sports and/or vigorous exercise in my free time). The validity of this scale has been assessed through a cross-cultural investigation, and the results indicated that it was valid for use in exercise settings for young people (Hagger et al., 2007). We selected the four items that appeared to best capture the supportive underlying intent and consequently convey as an informative meaning.

We then edited the wording of these 7-items to better reflect the work setting, as well as specifically focus on cash rewards. For example, to measure the controlling meaning, the original item “My coach tries to motivate me by promising to reward me if I do well” from the Controlling Coach Behavior Scale (Bartholomew et al., 2011) became “My boss tries to motivate me by promising to financially reward me if I do well,” whereas to measure the informative meaning, the original item “My PE teacher provides me with positive feedback when I do active sports and/or vigorous exercise in my free time” from the scale Perceived Autonomy Support Scale for Exercise Settings (Hagger and Chatzisarantis, 2007) became “My boss provides me with positive feedback when s/he gives me a cash reward.” In order to ensure content validity, we asked a panel of experts to review the edited items. The panel of experts was from different background, including researchers familiar with SDT and in scale construction, as well as practitioners specialized in compensation and reward programs. Finally, all items were translated using the back-translation procedure by Vallerand (1989) from English to French, and Greek, by a team of native bilinguals with proficient knowledge in the SDT literature. By validating the scale in three different languages—English, French, and Greek—we hoped to achieve a more universal application of this scale.

## Study 1

The goal of Study 1 was to test the factorial structure of the scale and assess whether it supported a two-factors structure reflecting the two distinct meanings, i.e., informative and controlling meaning, by showing how they were related to different types of motivation. Our initial test was to confirm a two-factor structure of the scale in the original language in which the previously existing scales had been developed, namely in English. To do so, we conducted an exploratory factor analyses (EFA) with our first set of English samples. Then, we subsequently analyzed the second English sample to replicate the factorial structure through confirmatory factor analyses (CFA) as well as investigate internal consistency. This test also allowed us to further test the convergent and divergent validity with motivation.

## Methodology

### Participants

#### Initial Sample

For the initial sample used to conduct EFA, 660 participants (41.3% men and 58.7% women) were recruited as part of a larger study. The participants all spoke English and were aged between 22 and 84 years old (mean = 50.7, SD = 10.6). A total of 92.2% had a bachelor’s degree or more in terms of education, 71.2% worked full-time, and 79.7% worked in the private sector. Average organizational tenure was 6.3 years (SD = 2.3).

#### Second Sample

For the second sample used to conduct CFA, 1045 participants (46.6% men and 53.4% women) were recruited in the context of another broader research program. The participants all spoke English and were aged between 24 and 73 years old (mean = 50.4, SD = 8.14) 92.2% had a bachelor’s degree or more in terms of education, 96.2% worked full-time, and % worked in the private sector. Average organizational tenure was 8.72 years (SD = 7.5). 76.82% were salaried workers.

### Procedure

Invitations to participate in the broader study were sent by email through an international consulting firm’s listserv. Participants received an invitation with a direct link to the online survey and completed the measures of interest for the present scale validation process, on a voluntary and anonymous basis and received no compensation for their participation.

### Measures

#### Motivation

In addition to completing the functional meaning of reward scale, participants in the second sample completed the Multidimensional Work Motivation Scale (MWMS; Gagné et al., 2015). Employees indicated the degree to which each item represented a reason why they chose to invest effort in their current job, using a 7-point Likert scale ranging from 1 (*Not at all*) to 7 (*Completely*). This included three items of the Intrinsic Motivation subscale (e.g., “Because I enjoy this work very much”), the three items of the Identified Motivation subscale (e.g., “Because this job fits my personal values”), the four items of the introjected motivation subscale (e.g., “Because I would feel ashamed if I did not succeed at this job”) and the six items of the Extrinsic Motivation subscale (e.g., “Because it allows me to make a lot of money”).

## Results and Discussion

### Exploratory Factor Analysis With Initial Sample

We used the principal axis method of estimation and promax rotation to do a set of EFA and gain a first insight in the structure of the items tapping into the functional meaning of cash rewards. Based on the eigenvalue criteria, two factors were retained in an EFA involving the 7 items. More specifically, all three items measuring the controlling meaning of cash rewards loaded on one factor and all four items measuring the informative meaning of cash rewards loaded on another factor. The controlling meaning factor explained 46.04% of the variance, and the informative meaning factor explained 33.54% of the variance, indicating that the two factors explained a total of 79.58% of the variance. The standardized factor loadings and item information for the two-factor model resulting from the EFA are provided in Table 1. These findings provided some initial evidence that the controlling and informative meaning of cash rewards constitute distinct dimensions of the functional meaning of cash rewards.

**TABLE 1:** Factor loading, items mean, and standard deviation of two-factors (EFA) for the three studies.

ITEMS	Study 1 (*n* = 660)			Study 2 (*n* = 304)			Study 3 (*n* = 260)		
	Loading	*M*	SD	Loading	*M*	SD	Loading	*M*	SD
**Controlling meaning of cash rewards**									
1. My boss tries to motivate me by promising to financially reward me if I do well.	0.651	2.78	2.12	0.773	3.57	2.02	0.862	2.25	1.70
2. The only reason my boss rewards me financially is to make me work harder.	0.921	2.98	2.16	0.832	2.70	1.78	0.838	2.30	1.71
3. My boss only uses cash rewards so that I stay focused on tasks during work.	0.917	2.81	2.14	0.854	2.59	1.61	0.832	2.08	1.51
**Informative meaning of cash rewards**									
4. My boss displays confidence in my ability to work when s/he gives me a cash reward.	0.863	4.03	2.09	0.792	4.32	1.98	0.919	2.61	1.88
5. My boss encourages me to work when s/he gives me a cash reward.	0.747	3.81	2.06	0.813	4.59	1.85	0.902	2.70	1.95
6. My boss provides me with positive feedback when s/he gives me a cash reward.	0.936	4.32	2.09	0.866	4.84	1.83	0.889	2.80	2.00
7. My boss cares about my work when s/he gives me a cash reward.	0.934	4.21	2.09	0.866	4.40	1.91	0.696	2.68	1.93

### Confirmatory Factor Analysis With Second English Sample

We then conducted CFA with robust maximum likelihood estimation, using Amos 18.0, to examine the construct validity for the functional meaning of cash rewards and test the adequacy of the initial two-factor structure found with the initial sample with the second sample. For the overall goodness-of-fit indexes, we used the Comparative Fit Index (CFI; Bentler, 1990), the Root Mean Square Error of Approximation (RMSEA; Hu and Bentler, 1999), the Incremental Fit Index (IFI; Bollen, 1989) and the Goodness of Fit Index (GFI; Jöreskog and Sörbom, 1989). Generally, values around 0.90 for the CFI, IFI, and GFI, and values below 0.08 for the RMSEA indicate a satisfactory fit.

Following Jöreskog’s (1993) method of alternative models, two alternative measurement models were tested. Model 1 (M1) assumed that only one factor underlies the observed variable and that the functional meaning of cash rewards is a unidimensional concept. In other words, this model would suggest that the functional meaning of monetary is a singular concept, without distinction between the controlling and informative meanings. Model 2 (M2) consisted of two latent variables representing the two hypothesized dimensions of the construct. This alternative model tested if the proposed dimensions (i.e., the controlling and informative meanings of cash rewards) explained the covariances of the observed variable. The fit for M1 proved to be inadequate: χ^2^ (df) = 125.192, df = 14; RMSEA = 0.345; GFI = 0.670; NFI = 0.707; IFI = 0.709; CFI = 0.709. The fit for M2 testing the two-factor model showed a significant improvement in the model-data fit over the M1 one-factor model: χ^2^ (df) = 3.844, df = 6; RMSEA = 0.052; GFI = 0.994; NFI = 0.996; IFI = 0.997; CFI = 0.997. We also computed the χ^2^/df to compare the models and since lower values generally indicate a better fit, found that M2 was more adequate χ^2^/df = 3.84, than M1, χ^2^/df = 125.19 (see Table 2). Factors’ loadings, items means and standard deviations for this sample are summarized in Table 1, while CFA results are presented in Table 3. Given that M1 showed overall superior model fit than M1, this further supported our hypothesis that the controlling and informative meanings of cash rewards are distinct dimensions of functional meanings (see Table 2).

**TABLE 2:** Results of the two competing models in Study 1 (*N* = 1,045).

Model	χ^2^ (df)	χ^2^/df	RMSEA	GFI	NFI	IFI	CFI
M1	1,752.69 (14)	125.19	0.34	0.67	0.71	0.71	0.71
M2	23.06 (6)	3.84	0.05	0.99	0.99	0.99	0.99

**TABLE 3:** Confirmatory factorial analyses for the three samples.

ITEMS	Study 1 (*n* = 1,045)		Study 2 (*n* = 304)		Study 3 (*n* = 260)	
	Factor 1	Factor 2	Factor 1	Factor 2	Factor 1	Factor 2
1. My boss tries to motivate me by promising to financially reward me if I do well.	0.922		0.886		0.853	
2. The only reason my boss rewards me financially is to make me work harder.	0.917		0.841		0.836	
3. My boss only uses cash rewards so that I stay focused on tasks during work	0.705		0.833		0.811	
4. My boss displays confidence in my ability to work when s/he gives me a cash reward.		0.924		0.878		0.912
5. My boss encourages me to work when s/he gives me a cash reward.		0.921		0.861		0.885
6. My boss provides me with positive feedback when s/he gives me a cash reward.		0.823		0.816		0.870
7. My boss cares about my work when s/he gives me a cash reward.		0.737		0.833		0.659

### Internal Reliability of the Scale

One critic often addressed to the controlling use of reward scale is its internal reliability deficiency. Indeed, Bartholomew et al. (2010) found the internal reliability of the controlling use of rewards to be quite low (α = 0.53). Moreover, in another paper, the reliability was moderate (α = 0.64; Karjane and Hein, 2015). The internal consistency for each scale in study 1 was reported in Table 4: the Cronbach’s alpha of controlling meaning and informative meaning are 0.88 and 0.92.

**TABLE 4:** Internal consistencies of the controlling meaning and informative meaning scores among the three studies.

Study	Controlling meaning	Informative meaning
Study 1	0.88	0.92
Study 2	0.77	0.87
Study 3	0.89	0.95

### Correlation With Motivation

In addition to establishing the adequacy of the factor structure of the newly developed measure of the function meaning of monetary rewards in the work setting, we also tested its relationships with work motivation. And the correlation results are shown in Table 5. Our hypothesis 1 was that, because cash rewards are in and of themselves an external factor, both informative and controlling meaning will be associated with external motivation. Consistent with our prediction, we found that the relationship between controlling and external motivation was significantly positive (*r* = 0.07, *p < 0.05); however, informative meaning was not associated with extrinsic motivation (*r* = 0.03, N.S.). One potential explanation is that, even though a cash reward is inherently an external factor, having an informative meaning may be a stronger factor enough to eliminate its association with extrinsic motivation. Our hypothesis 2 was that, since the underlying meaning of the cash rewards is more specifically relevant with regards to autonomous forms of motivation, informative meaning of cash rewards will be positively associated with autonomous type of motivation, whereas controlling meaning of cash rewards will be negatively associated with autonomous type of motivation. Consistent with our hypothesis, we found that the relationship between controlling and intrinsic motivation was significantly negative (*r* = −0.092, ^**^p < 0.01). The relationships between autonomous type of motivation, namely, identified motivation (*r* = 0.081, ^**^p < 0.01) and intrinsic motivation (*r* = 0.073, ^**^p < 0.01) were both significantly positively associated with informative meaning of cash rewards. These results provide support for our hypotheses 1 and 2: The two functional meanings (informative and controlling) are distinctive in that they are related to different types of motivation.

**TABLE 5:** Correlations and reliability coefficients along the diagonal for the variables in Study 1 (*N* = 1,045).

	1	2	3	4	5	6
1. Controlling meaning	(0.88)					
2. Informative meaning	0.60**	(0.92)				
3. Extrinsic motivation	0.07*	0.03	(0.68)			
4. Introjected motivation	0.03	0.02	0.27**	(0.78)		
5. Identified motivation	−0.06	0.08**	0.06	0.34**	(0.88)	
6. Intrinsic motivation	−0.09**	0.07**	−0.04	0.28**	0.84**	(0.92)

*p < 0.05; **p < 0.01.

## Study 2

The goal of Study 2 was to test and replicate the factorial structure with a French sample in order to validate the French translation of the scale. In addition to validating the scale in a different language, we further wanted to replicate findings from Study 1 showing the convergent and divergent validity and usefulness of the concept of the functional meaning in better understanding the motivational power of cash rewards.

## Methodology

### Participants

The sample was comprised of 304 adults (54.3% men and 45.7% women) working in the greater Montréal region. They all spoke French and were aged between 19 and 61 years old (mean = 34.9, SD = 10.1). From the sample, 59.1% had a bachelor’s degree or more in terms of education, 45.0% worked full-time. Average organizational tenure was 5.8 years (SD = 5.8), average job tenure was 3.1 years (SD = 3.5).

### Procedure and Measures

As in Study 1, invitations to participate in the broader study were sent by email through a provincial professional order’s listserv. Participants received an invitation with a direct link to the online survey and completed the measures of interest for the present scale validation process, namely the Functional meaning of cash reward scale and the French validated version of the Multidimensional Motivation at Work Scale (Gagné et al., 2015), on a voluntary and anonymous basis and received no compensation for their participation.

## Results and Discussion

### Confirmatory Factor Analysis

We conducted CFA to replicate the two-factorial structure obtained in Study 1. As in Study 1, we tested the two alternative models M1 proposing a single factor and M2 proposing two underlying factors reflecting the informative and the controlling meanings (see Table 6). Replicating the results of Study 1, M2 offered a superior and more adequate fit: χ^2^ (df) = 2.307, df = 14; RMSEA = 0.066; GFI = 0.981; NFI = 0.978; IFI = 0.987; CFI = 0.987, than M1: χ^2^ (df) = 19.409, df = 14; RMSEA = 0.246; GFI = 0.774; NFI = 0.714; IFI = 0.724; CFI = 0.722. This result thus provided additional support that the controlling and informative meanings of cash rewards constitute distinct and unique dimensions. Factors’ loadings, items means and standard deviations for this sample are summarized in Table 1, while CFA results are presented in Table 3.

**TABLE 6:** Results of the two competing models in Study 2 (*N* = 304).

Model	χ^2^ (df)	χ^2^/df	RMSEA	GFI	NFI	IFI	CFI
M1	271.73 (14)	19.41	0.25	0.77	0.71	0.72	0.72
M2	20.76 (9)	2.31	0.06	0.98	0.98	0.99	0.99

### Internal Consistency

Furthermore, as we previously did in Study 1, we examined the internal consistency analysis for each scale in Study 2. The internal consistency for each scale in Study 2 was reported in Table 2, the Cronbach’s alpha of controlling meaning and informative meaning are 0.77 and 0.87. In other words, the reliability of the scale seems acceptable in this second study.

### Correlations With Motivation

As in Study 1, we also tested the relationships among the functional meaning of monetary rewards and work motivations. The correlation results are shown in Table 7. Again, we found that the relationship between controlling and external motivation was significantly positive (*r* = 0.225, ^**^p < 0.01), but not between controlling and introjected motivation (*r* = 0.02, N.S.). Moreover, the relationship between informative and external motivation was significantly positive (*r* = 0.23, ^**^p < 0.01) as well as the relationship between information and introjected motivation (*r* = 16, ^**^p < 0.01). These results partially support hypothesis 1. Furthermore, the relationship between controlling and identified motivation was significantly negative (*r* = −0.148, ^**^p < 0.01), but the relationship between controlling and intrinsic motivation was not (*r* = −0.10, N.S.). The relationship between informative and identified motivation was significantly positive (*r* = 0.12, *p < 0.05), but the relationship between informative and intrinsic motivation was not (*r* = 0.08, N.S). These results partially support hypothesis 2.

**TABLE 7:** Correlations and reliability coefficients along the diagonal for the variables in Study 2 (*N* = 340).

	1	2	3	4	5	6
1. Controlling meaning	(0.77)					
2. Informative meaning	0.34**	(0.87)				
3. Extrinsic motivation	0.23**	0.23**	(0.83)			
4. Introjected motivation	0.02	0.16**	0.23**	(0.83)		
5. Identified motivation	−0.15**	0.12*	−0.19**	0.31**	(0.83)	
6. Intrinsic motivation	−0.10	0.08	−0.22**	0.17**	0.77**	(0.96)

*p < 0.05; **p < 0.01.

## Study 3

The goal was to test and replicate the factorial structure with a Greek sample in order to validate the Greek translation. As we did in Study 2, we aimed to further replicate findings in terms of the relation between the distinct meanings of cash rewards (i.e., informative and controlling) with specific types of motivation. Finally, an additional goal of Study 3 was to test the concurrent validity of the scale in relation to other relevant money-related constructs in the literature, namely, pay satisfaction, financial contingent self-worth and performance contingent rewards.

## Methodology

### Participants

The sample was comprised of 260 adults (31.9% men and 68.1% women) working in the greater Athens region. They all spoke Greek and were aged between 18 and 75 years old (mean = 37, SD = 15.6). From the sample, 78.4% had a bachelor’s degree or more in terms of education, 45.0% worked full-time. Average organizational tenure was 6.8 years (SD = 4.2), average job tenure was 7.6 years (SD = 7).

### Procedure and Measures

As in Studies 1 and 2, invitations to participate in the broader study were sent by email through a local consulting firm’s listserv. Participants received an invitation with a direct link to the online survey and participated in an anonymous and voluntary basis without receiving compensation for their participation. They completed the measures of interest for the present scale validation process, namely the Functional meaning of cash reward scale and the Greek version of the Motivation at Work Scale (Gagné et al., 2015). In addition to these measures, participants also completed Greek versions of the Pay Satisfaction Questionnaire (Heneman and Schwab, 1985), the Financial Self-Worth (Park et al., 2017) and the Performance Contingent Reward Scale (Houlfort et al., 2002).

#### Pay Satisfaction

Participants rated the extent to which they were satisfied with the pay, the raise and the benefits they receive at work, as well the way in which their pay is administered using the 18-item Pay Satisfaction Questionnaire (Heneman and Schwab, 1985). Each dimension of pay satisfaction is measured using 4 items (e.g., “Take-home pay”; “Benefit package”) using a 7-point scale ranging from 1 = very dissatisfied to 7 = very satisfied.

#### Financial Self-Worth

Employees’ financial self-worth was obtained using the 5-item measure of Contingent Financial Self-Worth Scale (e.g., “My self-esteem is influenced by how much money I make”; Park et al., 2017). Employees responded on a scale from 1 = Not at all important to 9 = Extremely important.

#### Performance Contingent Reward Scale

Employees’ perceptions of the financial incentives used at their workplace were assessed using the 12 items of the work-adapted version of the (e.g., “In my workplace, there are several cues and reminders indicating to me that I need to meet the standards set by organization if I want to get a bonus”; Houlfort et al., 2002). Participants rated the extent they agreed with each item using a 7-point Likert scale ranging from 1 = Strongly disagree to 7 = Strongly agree.

## Results and Discussion

### Confirmatory Factor Analysis

We conducted CFA to replicate the two-factorial structure obtained with the English and the French samples. As in Studies 1 and 2, we tested the two alternative models M1 proposing a single factor and M2 proposing two underlying factors reflecting the informative and the controlling meanings (see Table 8). Replicating the results of Study 1, M2 offered a superior and more adequate fit to the data: χ^2^ (df) = 5.145, df = 13; RMSEA = 0.127; GFI = 0.932; NFI = 0.962; IFI = 0.970; CFI = 0.969, than M1: χ^2^ (df) = 21.022, df = 14; RMSEA = 0.278; GFI = 0.726; NFI = 0.835; IFI = 0.841; CFI = 0.841. This result thus provided additional support that the controlling and informative meanings of cash rewards constitute distinct and unique dimensions. Factors’ loadings, items means and standard deviations for this sample are summarized in Table 1.

**TABLE 8:** Results of the two competing models in Study 3 (*N* = 260).

Model	χ^2^ (df)	χ^2^/df	RMSEA	GFI	NFI	IFI	CFI
M1	294.31 (14)	21.02	0.28	0.73	0.83	0.84	0.84
M2	66.88 (13)	5.14	0.12	0.93	0.96	0.97	0.97

### Internal Consistency

The internal consistency for each scale in Study 3 was reported in Table 4, the Cronbach’s alpha of controlling meaning and informative meaning are 0.89 and 0.95. Like we observed in Studies 1 and 2, these results indicate satisfactory internal consistency indices regarding the Functional Meaning of Cash Rewards Scale.

### Correlations With Motivation and Other Variables of Interest

We tested the relationships among the functional meaning of cash rewards and work motivations, in addition to the new variables. The correlation results are shown in Table 7. Again, consistent with our initial prediction, we found that the relationship between controlling and external motivation was significantly positive (*r* = 0.33, ^**^p < 0.01) as well as informative meaning and external motivation (*r* = 0.32, ^**^p < 0.01). Supporting our hypothesis 2, the relationship between controlling meaning and intrinsic motivation was significantly negative (*r* = −0.145, *p < 0.05), while the relationship between informative meaning and intrinsic motivation was significantly positive (*r* = 0.15, ^**^p < 0.01). Moreover, we found that controlling meaning was significantly and positively associated with pay satisfaction raise (*r* = 0.23, ^**^p < 0.01) and pay satisfaction structure (*r* = 0.18, ^**^p < 0.01), but not pay satisfaction level and benefits (Table 9). However, informative meaning was significantly associated with all four dimensions of the pay satisfaction, such that informative meaning was positively related to pay satisfaction raise (*r* = 0.23, ^**^p < 0.01), pay satisfaction structure (*r* = 0.23, ^**^p < 0.01), pay satisfaction level (*r* = 0.13, *p < 0.05), and pay satisfaction benefits (*r* = 0.18, ^**^p < 0.01). This provides support for our hypothesis 3a. In hypothesis 3b, we predicted that functional meaning of cash rewards will be associated with contingent financial reward and financial contingent self-worth even more strongly than its relationship to pay satisfaction. Supporting this hypothesis, we found the relationship between controlling and financial contingent self-worth (*r* = 0.304, ^**^p < 0.01) and performance contingent reward (*r* = 0.559, ^**^p < 0.01) to be significantly positive, even more so than its relationship to pay satisfaction. Consistently, the relationship between informative meaning and financial contingent self-worth (*r* = 0.275, ^**^p < 0.01), contingent reward (*r* = 0.544, ^**^p < 0.01) were significantly positive.

**TABLE 9:** Correlations and reliability coefficients along the diagonal for the variables in Study 3 (*N* = 260).

	1	2	3	4	5	6	7	8	9	10	11	12
1. Controlling meaning	(0.88)											
2. Informative meaning	0.68**	(0.95)										
3. Extrinsic motivation	0.33**	0.32**	(0.86)									
4. Introjected motivation	0.10	0.12*	0.37**	(0.78)								
5. Identified motivation	−0.01	0.13*	0.19**	0.48**	(0.92)							
6. Intrinsic motivation	−0.16*	0.15*	0.10	0.33**	0.66**	(0.89)						
7. Pay satisfaction – level	0.08	0.13*	0.07	0.01	0.15*	0.09	(0.95)					
8. Pay satisfaction – benefits	0.12	0.18**	0.07	0.02	0.10	0.05	0.80**	(0.92)				
9. Pay satisfaction – raise	0.23**	0.23**	0.18**	0.06	0.11	0.03	0.74**	0.76**	(0.84)			
10. Pay satisfaction – structure	0.18**	0.23**	0.15*	0.15*	0.18**	0.10	0.70**	0.69**	0.80**	(0.91)		
11. Financial contingent self-worth	0.30**	0.28**	0.38**	0.11	0.10	0.02	0.08	0.09	0.14*	0.11	(0.59)	
12. Performance contingent reward	0.56**	0.54**	0.30**	0.11	0.10	0.02	0.50**	0.52**	0.54**	0.52**	0.21**	(0.84)

*p < 0.05; **p < 0.01.

## General Discussion

As an empirically based approach to human motivation, from the start, SDT has evolved with a keen interest and desire to test, expand, and refine its propositions and integrate important new contributions into the framework. Perhaps the most controversial set of the findings within the umbrella of SDT is directly related to pay—namely, the findings concerning reward effects on intrinsic motivation and related concepts. Even though contingency rewards have been initially represented as an antecedent of controlled motivation (Gagné and Deci, 2005), prior research demonstrated that studying the motivational impacts of the reward itself is insufficient, it is rather the meaning (i.e., informative or controlling) associated with contingency rewards that could influence employees’ level of need satisfaction and work motivation (e.g., Thibault-Landry et al., 2019b). Nevertheless, even though research on SDT should continue to investigate the contextual nature of rewards, the research did not find yet adequate validated tools to do so (Forest et al., 2022). In the same vein, our current study contributes in the discussion of how different kind of rewards’ provision is linked both with different kinds beyond extrinsic and intrinsic) of motivation (Van den Broeck et al., 2021) as well as different motivational profiles in the work place (Howard et al., 2021).

In this paper, following the call by recent articles (e.g., Deci et al., 1999) to examine how tangible rewards and pay affect internalization of regulations for work behaviors and relate the functional significance of various pay contingencies to motivations and work outcomes, we validated a new scale (i.e., the Functional Meaning of Cash Rewards Scale) elaborated from SDT to assess the functional meaning of cash rewards offered in the workplace. The psychometric instrument was constructed based on two validated scales: the Controlling Use of Rewards subscale of the Controlling Coach Behavior Scale (Bartholomew et al., 2011) and Perceived Autonomy Support Scale for Exercise Settings (Hagger and Chatzisarantis, 2007). The two-factorial structure of the scale was replicated in a series of three studies, measuring respectively workplace cash rewards’ informative and controlling meanings. In Study 1, the English version of the scale was validated by exploring and then confirming its two-factor structure with two English-speaking employee samples. The two-factor structure was further replicated in a French-speaking employee sample of employees in Study 2 and in a Greek-speaking employees sample in Study 3, allowing us to validate its French and Greek version. Results from our three studies how distinct meanings attributed to cash rewards, i.e., informative or controlling, relate differently to autonomous, and controlled forms of motivation based on SDT.

More precisely, our results are in line with prior research on compensation describing the impact of contingency cash rewards on employees’ motivation in organizations (e.g., see Thibault-Landry et al., 2017; Thibault-Landry et al., 2019b; Olafsen and Deci, 2020). In this paper, using the Functional Meaning of Cash Rewards Scale, we demonstrated that using the monetary rewards perceived as informational led to healthier forms of motivation, greater psychological health, and better overall work intentions than did cash rewards perceived as controlling, because informational rewards are conducive to greater basic psychological need satisfaction. These and other findings suggest that rewards can have a distinct effect on individuals’ motivation and performance depending on whether they take on a need supportive or controlling meaning (Olafsen and Deci, 2020).

Moreover, some practical implications of this research must be acknowledged, especially those for the workplace. Mainly, the results of this research highlight the importance of considering compensation as a tool to stimulate employees’ optimal functioning, thus demystifying the taboo of using monetary rewards to influence the quality of work motivation. More specifically, this research has the potential to help understand why compensation and reward programs often fail to positively motivate workers or to elicit better performance from employees. Our study corroborates the latest data suggesting that most employers motivate their employees sub-optimally, using direct extrinsic motivators like money and prizes as enticement, or “incentives,” for future actions, rather than as indirect “rewards” that convey appreciation and recognition *after* a desired behavior has been achieved (Risher, 2013, 2015; Cerasoli et al., 2014; Cleveland et al., 2015). The Meaning of Reward Scale enables both academics and organizations to conduct research assessing employees’ perceptions of the rewards they receive at work. Along with other research, our scale contributes to the idea that employees seek more than money and aims at better understanding what employees want and why (Giancola, 2014). To this point, the research conducted to validate this scale indicates that rewards must be imbued with meaning, purpose, appreciation and intention in order to avoid feeling like empty gestures or mere transactions (Gagné and Forest, 2008; Shaffer and Arkes, 2009; Greene, 2014; Moller and Deci, 2014).

### Limitations and Future Studies

Despite its contribution to the concern of using contingency rewards in the workplace, this study exhibits some limitations, especially the fact that its results are based on cross-sectional study designs. Thus, common method bias (CMB) might have tainted our findings considering that the data was self-reported. However, it would have been challenging to assess several variables used in this study with other procedures (psychological experiences and states such as work motivation and meaning of cash rewards), and perhaps less precise (Spector, 2006). Moreover, two statistical procedures were executed to reduce CMB. First, in the English, French, and Greek sample, an additional factor was added while performing an additional CFA. Although fit indexes slightly increased, very small changes were observed related to factor loadings (Johnson et al., 2011). Second, a Harman’s-factor test (Fuller et al., 2016) was performed and also found that CMB was not an issue. However, further research could, for example, examine whether the meaning of cash rewards put forward in this paper may also lead to positive organizational outcomes such as increasing performance ratings and financial returns.

Second, the present study did not explore the predictive validity of the scale, since all analyses were based on cross-sectional associations. Future studies may further examine temporal stability of the measurement scale and causal relations between the meaning of cash rewards and its consequences by means of longitudinal or experimental studies. In addition, diary studies can be used to focus on intra-individual differences regarding the meaning of cash rewards and its correlates.

Finally, although the present paper validated the proposed scale in three languages (i.e., English, French, and Greek), more efforts need to be invested to support the external validation of the tool. For instance, the MWMS (Gagné et al., 2015) is now available in 25 different languages, namely English, French, Spanish, Portuguese, Dutch, Italian, Greek, Norwegian, Swedish, Finnish, Polish, German, Estonian, Croatian, Czech, Romanian, Turkish, Russian, Arabic, Persian/Farsi, Hebrew, Indonesian, Vietnamese, Japanese, and Chinese. The above raises possible doubts on the external validity of the proposed scale in this study and future research should continue to validate the scale in other populations/languages.

## Conclusion

The current findings contribute to the debate surrounding the motivational impact of monetary rewards and suggest that using such rewards is not inherently detrimental or beneficial. Instead, it would appear that it is the meaning that is conveyed through the presentation of the monetary rewards that is related to individuals’ motivation. Monetary rewards presented in a coercive, pressuring way risk conveying a controlling meaning. As such, they are more likely to be associated with more instrumental forms of motivation, such as extrinsic motivation, in which individuals focus on the external monetary gains. On the other hand, presenting monetary rewards in an encouraging and supportive way to convey an informative meaning can be a tool to contribute to individuals’ intrinsic motivation as they engage in the task, thus leading them to potentially perform and feel better. More research in the field is needed to investigate how monetary rewards are presented in the workplace, including how organizations present reward programs to their employees and to identify the best way to leverage monetary rewards to foster an informative meaning and intrinsic as well as identified motivation.

## Data Availability Statement

The original contributions presented in the study are included in the article/supplementary material, further inquiries can be directed to the corresponding author.

## Author Contributions

AT contributed to data collection, data analysis, theoretical background, and discussion. KP contributed to discussion, data collection, and theoretical background. M-AG contributed to methodology, data analysis, and discussion. JF contributed to discussion and theoretical background. All authors contributed to the article and approved the submitted version.

## Conflict of Interest

The authors declare that the research was conducted in the absence of any commercial or financial relationships that could be construed as a potential conflict of interest.

## Publisher’s Note

All claims expressed in this article are solely those of the authors and do not necessarily represent those of their affiliated organizations, or those of the publisher, the editors and the reviewers. Any product that may be evaluated in this article, or claim that may be made by its manufacturer, is not guaranteed or endorsed by the publisher.
